# Gender difference in preference of specialty as a career choice among Japanese medical students

**DOI:** 10.1186/s12909-016-0811-1

**Published:** 2016-11-10

**Authors:** Ryuichi Kawamoto, Daisuke Ninomiya, Yoshihisa Kasai, Tomo Kusunoki, Nobuyuki Ohtsuka, Teru Kumagi, Masanori Abe

**Affiliations:** 1 Department of Community Medicine, Ehime University Graduate School of Medicine, Toon-city, Ehime-ken 791-0295 Japan; 2 Department of Internal Medicine, Seiyo Municipal Nomura Hospital, 9-53 Nomura, Nomura-cho, Seiyo-city, Ehime-ken 797-1212 Japan

**Keywords:** Career choice, Gender difference, Japanese medical students

## Abstract

**Background:**

In Japan, the absolute deficiency of doctors and maldistribution of doctors by specialty is a significant problem in the Japanese health care system. The purpose of this study was to investigate the factors contributing to specialty preference in career choice among Japanese medical students.

**Methods:**

A total of 368 medical students completed the survey giving an 88.2 % response rate. The subjects comprised 141 women aged 21 ± 3 (range, 18–34) years and 227 men aged 22 ± 4 (range, 18–44) years. Binary Logistic regression analysis was performed using specialty preferences as the criterion variable and the factors in brackets as six motivational variables (e.g., Factor 1: educational experience; Factor 2: job security; Factor 3: advice from others; Factor 4: work-life balance; Factor 5: technical and research specialty; and Factor 6: personal reasons).

**Results:**

Women significantly preferred pediatrics, obstetrics & gynecology, and psychology than the men. Men significantly preferred surgery and orthopedics than the women. For both genders, a high odds ratio (OR) of “technical & research specialty” and a low OR for “personal reasons” were associated with preference for surgery. “Technical & research specialty” was positively associated with preference for special internal medicine and negatively for pediatrics. “Work-life balance” was positively associated with preference for psychology and negatively for emergency medicine. Among the women only, “technical & research specialty” was negatively associated with preference for general medicine/family medicine and obstetrics & gynecology, and “job security” was positively associated for general medicine/family medicine and negatively for psychology. Among men only, “educational experience” and “personal reasons” were positively, and “job security” was negatively associated with preference for pediatrics. For both genders, “work-life balance” was positively associated with preference for controllable lifestyle specialties.

**Conclusion:**

We must acknowledge that Japanese medical students have dichotomized some motivations for their specialty preference based on gender. Systematic improvements in the working environment are necessary to solve these issues.

## Background

In Japan, there have been absolute deficiencies in the number of physicians. This number of doctors belongs to the lowest group in the Organization for Economic Co-operation and Development (OECD) [1]. In addition to the absolute deficiency of doctors, maldistribution of doctors among the various specialties is a serious problem in the Japanese health care system. A Japanese governmental report revealed that obstetrics & gynecology, pediatrics, and anesthesiology have suffered a more severe shortage than other specialties [2]. Moreover, the decreasing tendency of doctors to choose internal medicine and surgery has accelerated [3].

In Japan, internal medicine showed the highest preference rate, followed by general surgery, pediatrics, and emergency medicine. The gender ratio varied according to the specialty [4], and the preference rates for general surgery, orthopedics, neurosurgery, and emergency medicine were significantly higher in men than in women, while those for obstetrics & gynecology, pediatrics, and dermatology were significantly higher in women [5]. Women now account for about one third to half of medical students and have become a growing part of medical schools in Japan as well as the U.S. and Europe [6, 7], however female physicians are still underrepresented in some specialties such as surgery and emergency medicine [8].

Choosing a specialty is a complex process and may be influenced by several confounding factors. Distribution of medical students’ career choices among specialties varies considerably. Recent studies in other countries identified several factors related to the choice and preference, such as gender, career opportunities, prestige, and income [9–11]. Enoch et al. demonstrated that specialties that feature a controllable lifestyle (control of work hours) were defined as anesthesiology, dermatology, emergency medicine, neurology, ophthalmology, otolaryngology, pathology, psychiatry, and radiology, while non-controllable lifestyle specialties were surgery, internal medicine, family practice, pediatrics, orthopedic surgery, and obstetrics & gynecology [12]. In the USA, interest in surgery, pediatrics, and obstetrics & gynecology has declined, but the popularity of controllable lifestyle fields such as radiology, psychiatry, dermatology, and ophthalmology has been increasing [13]. Controllable lifestyle is becoming an increasingly important factor in choice of specialty by medical students. Moreover, for surgical careers, the decision to have a family (e.g., children) was a more significant influence for women than men [14]. Thus, it is important to know the expectations of future physicians as they play a role in their career choice. There are many studies on career choice of medical students in Western countries. However, studies exploring the impact of gender differences regarding specialty preferences in career choice are still lacking in Japan.

The purpose of this study was to compare specialty preferences and the motivational factors of Japanese medical students. We investigated associations between motivational factors and specialty preference in order to determine the factors contributing to specialty preferences as a career.

## Methods

### Participants

We cross-sectionally conducted a survey of 1^st^ to 5^th^year medical students (*N* = 450) from one Japanese regional university school of medicine. From April 2009 to 2013 (in Japan, the academic year begins in April), the self-administered questionnaire was distributed and collected in class within the first 4 weeks of the start of medical school. Individual responses required a signature (anonymous responses were also permitted), and completion of the questionnaire was voluntary. The study was approved by the ethics committee of the Ehime University Graduate School of Medicine (IRB: 1507004), and informed consent was obtained from all subjects.

### Questionnaire

We used a modified questionnaire enquiring about their specialty preference and to what extent their decision was influenced by a set of given criteria that were developed by Takeda et al. [15]. Questions on gender, age, academic year, admission from hometown, type of high school, admission by a special policy aimed directly at increasing the number of rural physicians as the main purpose (chiiki-waku in Japanese), student preparing for the entrance exam next year, presence of medical relatives, and growing up in a rural area were included.

Participants were asked to specify which of the following 14 medical specialties they intended to pursue: general medicine/family medicine (GM/FM), internal medicine, surgery, paediatrics, obstetrics & gynaecology, psychiatry, anaesthesiology, emergency medicine, dermatology, orthopaedics, ophthalmology, otolaryngology, urology and radiology, or “other”. They were instructed to choose their most preferred specialty and other specialties ‘under consideration’ with no restriction in the number. When “other” was chosen for a non-listed specialty, respondents were asked to specify which discipline they were choosing. In Japan, non-controllable lifestyle specialties are general medicine/family practice, special Internal medicine, surgery, pediatrics, obstetrics & gynecology, anesthesiology, emergency medicine, and orthopedic surgery, and controllable lifestyle specialties are psychiatry, dermatology, ophthalmology, otolaryngology, urology, and radiology [13, 16]. With regard to choice of a specialty, we [17] previously defined the following 6 factors based on the items that were grouped together: Factor 1: educational experience; Factor 2: job security; Factor 3: advice from others; Factor 4: work-life balance; Factor 5: technical and research specialty; and Factor 6: personal reasons. These 6 factors collectively accounted for 47.6 % of the variance in responses. We calculated Cronbach’s alpha coefficiencies which demonstrated internal consistency that ranged between 0.55 and 0.84. The subscale response to the influences were rated on a 4-point likert scale (1 = not at all, 2 = not particularly, 3 = fairly well, and 4 = extremely well).

### Data analysis

Statistical analysis was performed using IBM SPSS Statistics Version 21 (Statistical Package for Social Science, Chicago, IL, USA). Data are presented as the mean ± standard deviation (SD) unless otherwise specified. Gender differences in the subscales by level of interest in choice of a specialty as a career were examined using Mann-Whitney U test for continuing variables or χ^2^-test for categorical variables. Finally, Binary Logistic regression analysis was performed in order to derive confounding factors associated with level of interest in choosing a specialty as a career by gender. The interactive effect of gender and confounding factors was evaluated using a general linear model. A *p*-value <0.05 was considered significant.

## Results

### Characteristics of female and male students

Of 417 students from whom the questionnaire could be collected, 368 completed the survey giving an 88.2 % response rate. The subjects comprised 141 women aged 21 ± 3 (range, 18–34) years and 227 men aged 22 ± 4 (range, 18–44) years (Table 1). “Student preparing for the entrance exam next year” ranked higher in men than in women and “having a role model” ranked higher in women than in men. There were no inter-group differences regarding age, academic year, admission from home, public high school graduation, combined junior high and high school graduation, admission by a special policy, presence of medical relatives, and growing up in a rural area.

**Table 1:** Characteristics of female and male medical students

	Total	Women	Men	*P*-value
Characteristics	*N* = 368	*N* = 141	*N* = 227	
Age, years	21 ± 4	21 ± 3	22 ± 4	0.258
Academic year				
1^st^-2^nd^	179 (48.6)	68 (48.2)	111 (48.9)	0.915
3^rd^-5^th^	189 (51.4)	73 (51.8)	116 (51.1)	
Admission from hometown				
Yes	168 (45.7)	64 (45.4)	104 (45.8)	1.000
No	200 (54.3)	77 (54.6)	123 (54.2)	
Public high school graduation				
Yes	195 (53.0)	76 (53.9)	119 (52.4)	0.830
No	173 (47.0)	65 (46.1)	108 (47.6)	
Combined junior high and high school graduation				
Yes	164 (44.6)	64 (45.4)	100 (44.1)	0.830
No	204 (55.4)	77 (54.6)	127 (55.9)	
Admission by a special policy				
Yes	23 (6.3)	6 (4.3)	17 (7.5)	0.270
No	345 (93.8)	135 (95.7)	210 (92.5)	
Student preparing for the entrance exam next year				
Yes	175 (47.6)	55 (39.0)	120 (52.9)	**0.010**
No	193 (52.4)	86 (61.0)	107 (47.1)	
Presence of medical relatives				
Yes	162 (44.0)	63 (44.7)	99 (43.6)	0.914
No	206 (56.0)	78 (55.3)	128 (56.4)	
Growing up in a rural area				
Yes	47 (12.8)	19 (13.5)	28 (12.3)	0.750
No	321 (87.2)	122 (86.5)	199 (87.7)	
Presence of a role model				
Yes	152 (41.3)	67 (47.5)	85 (37.4)	**0.064**
No	216 (58.7)	74 (52.5)	142 (62.6)	

Data are presented as number (%) or mean ± standard deviation. *P*-value from Mann-Whitney U-test for continuing variables and χ^2^-test for categorical variables. Bold numbers indicate significance (*p* < 0.05)

### Female and male students’ career preferences

Table 2 shows the levels of interest for specialty choices as a career by female and male students. The most common specialties chosen by the students were general medicine/family medicine (18.8 %), surgery (9.5 %), pediatrics (7.6 %), special Internal medicine (7.1 %), emergency medicine (4.6 %), orthopedics (3.8 %), and urology (3.3 %). The other specialties chosen are listed in Table 2. Women significantly preferred pediatrics, obstetrics & gynecology, and psychology than men. Men significantly preferred surgery and orthopedics than women.

**Table 2:** Female and male students’ career preferences

		Total	Women	Men	*P*-value
Specialty preference	Choice	*N* = 368	*N* = 141	*N* = 227	
General medicine/Family medicine	First	69 (18.8)	27 (19.1)	42 (18.5)	0.695
	Second	156 (42.4)	63 (44.7)	93 (41.0)	
Special Internal medicine	First	26 (7.1)	6 (4.3)	20 (8.8)	0.172
	Second	82 (22.3)	29 (20.6)	53 (23.3)	
Surgery	First	35 (9.5)	6 (4.3)	29 (12.8)	**<0.001**
	Second	113 (30.7)	33 (23.4)	80 (35.2)	
Pediatrics	First	28 (7.6)	12 (8.5)	16 (7.0)	**0.038**
	Second	102 (27.7)	49 (34.8)	53 (23.3)	
Obstetrics & Gynecology	First	11 (3.0)	10 (7.1)	1 (0.4)	**<0.001**
	Second	44 (12.0)	28 (19.9)	16 (7.0)	
Emergency medicine	First	17 (4.6)	4 (2.8)	13 (5.7)	0.436
	Second	96 (26.1)	37 (26.2)	59 (26.0)	
Psychology	First	8 (2.2)	4 (2.8)	4 (1.8)	**0.038**
	Second	48 (13.0)	26 (18.4)	22 (9.7)	
Anesthesiology	First	6 (1.6)	4 (2.8)	2 (0.9)	0.351
	Second	46 (12.5)	17 (12.1)	29 (12.8)	
Orthopedics	First	14 (3.8)	0	14 (6.2)	**<0.001**
	Second	69 (18.8)	10 (7.1)	59 (26.0)	
Dermatology	First	3 (0.8)	2 (1.4)	1 (0.4)	0.112
	Second	32 (8.7)	17 (12.1)	15 (6.6)	
Ophthalmology	First	6 (1.6)	3 (2.1)	3 (1.3)	0.531
	Second	30 (8.2)	9 (6.4)	21 (9.3)	
Otolaryngology	First	4 (1.1)	0 (0)	4 (1.8)	0.196
	Second	26 (7.1)	8 (5.7)	18 (7.9)	
Urology	First	12 (3.3)	3 (2.1)	9 (4.0)	0.385
	Second	0	0	0	
Radiology	First	5 (1.4)	4 (2.8)	1 (0.4)	0.053
	Second	23 (6.3)	12 (8.5)	11 (4.8)	
Others	First	1 (0.3)	1 (0.7)	0	0.350
	Second	8 (2.2)	4 (2.8)	4 (1.8)	

Data are presented as numbers (%). *P*-value from χ^2^-test. Bold numbers indicate significance (*p* < 0.05)

### Rating of motivational factors for specialty preference

Table 3 shows female and male motivational factors for specialty preference. Compared with men, women considered “memorable experience in a class or clinical rotation”, “encounter with role model teachers”, “advice from teachers/consultants”, and “interest in targeted populations such as children or the elderly” to be more important reasons for choosing a specialty, and “expected income” to be less important.

**Table 3:** Rating of female and male motivational factors for specialty preference

	Total	Women	Men	
	*N* = 368	*N* = 141	*N* = 227	*P-value*
Factor 1: Educational experience	3.0 ± 0.6	3.1 ± 0.7	3.0 ± 0.6	**0.009**
15) Received excellent teachings	3.1 ± 0.7	3.2 ± 0.7	3.1 ± 0.7	0.111
14) Memorable experience in a class or clinical rotation	3.0 ± 0.7	3.2 ± 0.8	2.9 ± 0.7	**0.005**
16) Comfortable atmosphere at the specialty department	3.1 ± 0.8	3.2 ± 0.8	3.1 ± 0.7	0.268
17) Encounter with role model teachers	2.9 ± 0.9	3.1 ± 0.9	2.8 ± 0.9	**0.024**
Factor 2: Job security	2.1 ± 0.6	2.1 ± 0.5	2.1 ± 0.6	0.392
19) Advice/Expectation of parents	2.2 ± 0.9	2.3 ± 0.9	2.2 ± 0.9	0.178
26) Expected income	2.3 ± 0.8	2.1 ± 0.7	2.4 ± 0.9	**0.002**
24) Ease of opening practice	2.0 ± 0.8	1.9 ± 0.7	2.1 ± 0.8	0.077
25) Expectation to inherit practice of my parents/relatives	1.6 ± 0.8	1.5 ± 0.7	1.7 ± 0.9	0.121
23) Job availability	2.5 ± 0.8	2.6 ± 0.8	2.5 ± 0.9	0.250
Factor 3: Advice from others	2.1 ± 0.7	2.1 ± 0.8	2.1 ± 0.7	0.175
20) Advice from senior students/residents	2.1 ± 0.9	2.2 ± 0.9	2.0 ± 0.8	0.154
21) Advice from teachers/consultants	2.2 ± 0.9	2.4 ± 1.0	2.2 ± 0.9	**0.037**
22) Influence of friends	2.0 ± 0.8	1.9 ± 0.7	2.0 ± 0.8	0.578
Factor 4: Work-life balance	2.4 ± 0.7	2.5 ± 0.7	2.4 ± 0.7	0.087
27) Working hours	2.4 ± 0.9	2.5 ± 0.9	2.3 ± 0.9	0.080
28) Attainable lifestyle	2.7 ± 0.9	2.8 ± 0.9	2.7 ± 0.9	0.106
30) Risk of my malpractice law suits	2.2 ± 0.8	2.2 ± 0.8	2.2 ± 0.8	0.587
Factor 5: Technical & research specialty	3.0 ± 0.6	3.0 ± 0.6	3.0 ± 0.6	0.766
5) Interest in the surgical procedures or technologies	3.1 ± 0.8	3.0 ± 0.9	3.1 ± 0.8	0.338
6) Mastering the specialty	3.0 ± 0.8	2.9 ± 0.8	3.0 ± 0.7	0.298
4) Interest in the research or scientific aspects	3.0 ± 0.8	3.0 ± 0.8	3.0 ± 0.8	0.884
2) Interest in the organ specialty	3.0 ± 0.8	3.0 ± 0.8	3.0 ± 0.8	0.377
Factor 6: Personal reasons	2.5 ± 0.6	2.6 ± 0.6	2.5 ± 0.7	0.290
11) I suffering(ed) from an illness of the specialty	2.2 ± 1.0	2.2 ± 1.0	2.3 ± 1.1	0.520
12) Friend/family suffers(ed) from an illness of the specialty	2.2 ± 1.0	2.5 ± 1.0	2.4 ± 1.1	0.401
13) Became interested in the specialty before medical school	2.5 ± 0.9	2.6 ± 0.9	2.5 ± 0.9	0.124
3) Interest in the targeted populations such as children or the elderly	2.9 ± 0.8	3.0 ± 0.8	2.8 ± 0.8	**0.010**

Data are presented as mean ± standard deviation. *P*-value from Mann-Whitney U test. Bold numbers indicate significance (*p* < 0.05)

### Motivational factors associated with specialty preference

Table 4 shows the similarities and differences in both genders regarding motivational factors and specialty preference after adjusting other characteristics. For both genders, a high odds ratio (OR) for the “technical & research specialty”, but a low OR for “personal reasons” were associated with preference for surgery. “Technical & research specialty” was positively associated with preference for special internal medicine and negatively for pediatrics. “Work-life balance” was positively associated with preference for psychology and negatively for emergency medicine. It was only among women that “technical & research specialty” was negatively associated with preference for general medicine/family medicine and obstetrics & gynecology, and “job security” was positively associated with general medicine/family medicine, but negatively with psychology. It was only among men that “educational experience” and “personal reasons” were positively associated, but “job security negatively associated with preference for pediatrics. When gender as an interaction term was studied in the association between motivational factors and preference of specialty, two significant differences were found. Each low ORs of “technical & research specialty” for general medicine/family medicine and “educational experience” for anesthesiology had a stronger association with female than male preference.

**Table 4:** Motivational factors associated with specialty preference by gender

	Odds ratio (95 % CI)		Gender interaction
	Women	Men	*p*-value
Specialty preference	*N* = 141	*N* = 227	
General medicine/Family medicine (Yes/No)			
Job security	**2.21 (1.04–4.69)**	------	0.104
Technical & research specialty	**0.29 (0.14–0.59)**	------	**0.022**
Special Internal medicine (Yes/No)			
Technical & research specialty	**2.01 (1.10–3.68)**	**2.28 (1.32–3.94)**	0.737
Surgery (Yes/No)			
Work-life balance	0.54 (0.28–1.06)	------	0.373
Technical & research specialty	**3.41 (1.39–8.35)**	**2.94 (1.70–5.10)**	0.732
Personal reasons	**0.18 (0.07–0.44)**	**0.49 (0.31–0.79)**	0.276
Pediatrics (Yes/No)			
Educational experience	------	**1.94 (1.16–3.25)**	0.060
Job security	------	**0.52 (0.31–0.85)**	0.345
Technical & research specialty	**0.44 (0.24–0.82)**	**0.55 (0.32–0.93)**	0.616
Personal reasons	------	**1.66 (1.01–2.73)**	0.207
Obstetrics & Gynecology (Yes/No)			
Educational experience	0.56 (0.29–1.07)	------	0.154
Advice from others	1.69 (0.91–3.14)	------	0.175
Technical & research specialty	**0.44 (0.22–0.89)**	------	0.512
Psychology (Yes/No)			
Job security	**0.19 (0.04–0.80)**	------	0.135
Advice from others	**2.45 (1.10–5.46)**	------	0.067
Work-life balance	**3.01 (1.22–7.43)**	**1.88 (1.02–3.49)**	0.537
Personal reasons	2.06 (0.93–4.56)	------	0.571
Anesthesiology (Yes/No)			
Educational experience	**0.32 (0.15–0.67)**	------	**0.032**
Technical & research specialty	------	1.81 (0.91–3.62)	0.412
Emergency medicine (Yes/No)			
Job security	------	0.55 (0.29–1.02)	0.070
Advice from others	------	**1.83 (1.09–3.09)**	0.152
Work-life balance	**0.46 (0.23–0.90)**	**0.53 (0.32–0.89)**	0.410
Technical & research specialty	2.19 (0.98–4.90)	------	0.090
Personal reasons	**0.45 (0.21–0.97)**	**0.61 (0.38–0.98)**	0.903
Orthopedics (Yes/No)			
Educational experience	**0.21 (0.08–0.57)**	------	0.122
Job security	------	2.17 (0.96–4.88)	0.885
Advice from others	2.30 (0.85–6.25)	------	0.070
Work-life balance	**4.87 (1.63–14.6)**	------	0.227

Yes, first and second choice. Data were adjusted for all confounding factors in Tables 1 and 3 by Binary Logistic regression analysis, and presented regarding motivational factors as the final model. Bold numbers indicate significance (*p* < 0.05)

*CI* confidence interval

### Motivational factors associated with controllable and non-controllable lifestyle groups of specialties by gender

For both genders, “work-life balance” was positively associated with preference for controllable lifestyle specialties (Table 5). Among women only, a high OR of “advice from others”, but a low OR of “educational experience” was associated with preference for controllable lifestyle specialties.

**Table 5:** Motivational factors associated with controllable and non-controllable lifestyle groups of specialties by gender

	Odds ratio (95 % CI)		Gender Interaction
	Women	Men	*p*-value
Specialty preference	*N* = 141	*N* = 227	
Non-controllable lifestyle group (Yes/No)			
Technical & research specialty	0.16 (0.02–1.53)	------	0.156
Personal reasons	0.15 (0.02–1.35)	------	0.783
Controllable lifestyle group (Yes/No)			
Educational experience	**0.45 (0.23–0.88)**	0.67 (0.42–1.06)	0.683
Job security	0.36 (0.13–1.01)	------	0.104
Advice from others	**2.24 (1.11–4.54)**	------	0.105
Work-life balance	**4.27 (1.94–9.40)**	**1.84 (1.24–2.73)**	0.056
Technical & research specialty	0.56 (0.29–1.09)	------	0.099

Non-controllable lifestyle group of specialties consisted of general medicine/family practice, special Internal medicine, surgery, pediatrics, obstetrics & gynecology, anesthesiology, emergency medicine, and orthopedics, and controllable lifestyle were defined as psychiatry, dermatology, ophthalmology, otolaryngology, urology, and radiology. Yes, first and second choice. Data were adjusted for all confounding factors in Tables 1 and 3 by Binary Logistic regression analysis, and presented regarding motivational factors as the final model. Bold numbers indicate significance (*p* < 0.05)

*CI* confidence interval

## Discussion

The purpose of this study was to investigate and compare male and female preferences for specialties and motivational factors among Japanese medical students. Our results showed some gender differences in choice of specialty by the medical students. Women considered “interest in targeted populations such as children or the elderly” to be a more important factor for choosing a specialty, and “expected income” to be less important. Moreover, they significantly preferred pediatrics, obstetrics & gynecology, and psychology than men. Men significantly preferred surgery and orthopedics. There were some differences between the genders regarding motivational factors associated with choice of specialty. Lastly, both the male and female students who preferred the controllable lifestyle specialties considered work-life balance to be important.

Our findings provide some important insights in medical student’s choice of specialty. In Japan, Fukuda et al. [5] reported that internal medicine has the highest preference rate followed by general surgery, pediatrics, and emergency medicine, and the preference rates for general surgery, orthopedics, neurosurgery, and emergency medicine were significantly higher in men than in women, while those for obstetrics & gynecology, pediatrics, and dermatology were higher in women. Gender differences in choice and preference of specialty among medical students are common internationally, and previous investigations in other countries also demonstrated that pediatrics and obstetrics & gynecology are predominantly preferred by women, surgery by men, and internal medicine is pursued by both genders [14, 18, 19]. Similar patterns were observed in our study, and gender-related specialty preferences were apparent. The fact that more women than men preferred obstetrics & gynecology and pediatrics could be explained by the high number of female physicians who are in these specialties and this has been the trend over the past decades. Hence, there are more same gender role models [20]. In Sweden, however, gender similarities among medical students regarding specialty preference are striking and contrast with research from other western countries [20]. Among Saudi undergraduate medical students, most women preferred surgery, followed by pediatrics and ophthalmology [19]. Individual preferences change extensively over time. Workload in these specialties is expected to increase as the number of female doctors increase due to changes in life circumstances such as pregnancies and child-rearing.

Women and men differed somewhat regarding the importance placed on the six motivational factors. Women rated “educational experience” (e.g., “memorable experience in a class” or “clinical rotation and “encounter with role model teachers”), “advice from teachers/consultants”, and “interest in targeted populations such as children or the elderly” higher, and “expected income” lower than men. Women tended to place a higher value on comprehensive patient care and existence of role models than men in choice of specialty. This is consistent with past work [10, 21, 22] indicating that women are more relationship-oriented than men.

Aasland et al. [23] showed that medical students who prefer surgery or internal medicine were more motivated by medical challenges and career possibilities, while those who prefer psychiatry or general medicine are more motivated by conditions such as variety and time for own family. Prestige of a specialty was less emphasized after graduation while the optimal combination of an interesting job and a good private life increased [23].

For both genders, work-life balance was an important factor that contributed to students preferring specialties in the controllable life style group. Controllable lifestyle was found to be strongly associated with recent trends in choice of specialty for both genders, and could not be explained solely by specialty preferences of women [24]. Dorsey et al. showed that the percentage of women choosing specialties with a controllable lifestyle increased from 18 % in 1996 to 36 % in 2003 and the percentage for men grew from 28 to 45 % [24]. Students for whom work-life balance was extremely important (odds ratio [OR] = 0.6) were less likely to prefer surgery. Students for whom work-life balance (OR = 2.2) and continuity of care (OR = 2.1) were extremely important were more likely to prefer general practice [25]. Understanding that perceptions towards prestige and lifestyle differ among students may be useful when advising medical students about choice of specialty [26].

There are some limitations to this study. First, our cross-sectional study design does not eliminate potential causal relationships between the motivational factors of medical students and career choice. Second, this study involved a limited number of students who belong to one local university. Therefore, the demographics and referral source may limit generalizability. Third, as we used a self-administered questionnaire developed for 1st to 5th year medical students, some of the characteristics (such as motivational factors and choice of specialty) examined appeared to be more suitable for students in a higher academic year. Fourth, our study measured students’ interest in a career, but not their actual choice after graduation. Future research using longitudinal data collection will enable us to monitor the relationship between early stated interest and actual behavior.

## Conclusion

The present study showed that medical students have a positive and realistic picture of what choosing a specialty as a career involves. Women and men differed somewhat in the importance they put on about six motivational factors. We must acknowledge that Japanese medical students have dichotomized some motivations for their preference of specialty and that is based on gender. Gender differences and mismatch of choices influence the current and future maldistributin of specialties. In Japan, systematic improvements in the work environment are necessary to solve these issues.
